# Human iPSCs derived astrocytes rescue rotenone-induced mitochondrial dysfunction and dopaminergic neurodegeneration in vitro by donating functional mitochondria

**DOI:** 10.1186/s40035-020-00190-6

**Published:** 2020-04-24

**Authors:** Xiao-Yu Cheng, Sangita Biswas, Juan Li, Cheng-Jie Mao, Olga Chechneva, Jing Chen, Kai Li, Jiao Li, Jin-Ru Zhang, Chun-Feng Liu, Wen-Bin Deng

**Affiliations:** 1 Department of Neurology and Suzhou Clinical Research of Neurological Diseases, The Second Affiliated Hospital of Soochow University, Suzhou, 215004 China; 2 Department of Biochemistry and Molecular Medicine, University of California Davis, Sacramento, CA 95817 USA; 3 Shriners Hospital for Children of Northern California, Sacramento, CA 95817 USA; 4 School of Pharmacy, Ningxia Medical University, Yinchuan, 750004 China; 5 Key Laboratory of Hui Medicine Modernization, Ministry of Education, Yinchuan, 750004 China

**Keywords:** Parkinson’s disease, iPSCs, Dopaminergic neurons, Rotenone, Astrocytes, Mitochondrial transfer, p38, Neuroprotection, Mitochondrial disease

## Abstract

**Background:**

Parkinson’s disease (PD) is one of the neurodegeneration diseases characterized by the gradual loss of dopaminergic (DA) neurons in the substantia nigra region of the brain. Substantial evidence indicates that at the cellular level mitochondrial dysfunction is a key factor leading to pathological features such as neuronal death and accumulation of misfolded α-synuclein aggregations. Autologous transplantation of healthy purified mitochondria has shown to attenuate phenotypes in vitro and in vivo models of PD. However, there are significant technical difficulties in obtaining large amounts of purified mitochondria with normal function. In addition, the half-life of mitochondria varies between days to a few weeks. Thus, identifying a continuous source of healthy mitochondria via intercellular mitochondrial transfer is an attractive option for therapeutic purposes. In this study, we asked whether iPSCs derived astrocytes can serve as a donor to provide functional mitochondria and rescue injured DA neurons after rotenone exposure in an in vitro model of PD.

**Methods:**

We generated DA neurons and astrocytes from human iPSCs and hESCs. We established an astroglial-neuronal co-culture system to investigate the intercellular mitochondrial transfer, as well as the neuroprotective effect of mitochondrial transfer. We employed immunocytochemistry and FACS analysis to track mitochondria.

**Results:**

We showed evidence that iPSCs-derived astrocytes or astrocytic conditioned media (ACM) can rescue DA neurons degeneration via intercellular mitochondrial transfer in a rotenone induced in vitro PD model. Specifically, we showed that iPSCs-derived astrocytes from health spontaneously release functional mitochondria into the media. Mito-Tracker Green tagged astrocytic mitochondria were detected in the ACM and were shown to be internalized by the injured neurons via a phospho-p38 depended pathway. Transferred mitochondria were able to significantly reverse DA neurodegeneration and axonal pruning following exposure to rotenone. When rotenone injured neurons were cultured in presence of ACM depleted of mitochondria (by ultrafiltration), the neuroprotective effects were abolished.

**Conclusions:**

Our studies provide the proof of principle that iPSCs-derived astrocytes can act as mitochondria donor to the injured DA neurons and attenuate pathology. Using iPSCs derived astrocytes as a donor can provide a novel strategy that can be further developed for cellular therapy for PD.

## Background

Parkinson’s disease (PD) is a progressive nervous system disorder that affects movement. Parkinson’s symptoms include resting tremor, bradykinesia, rigidity, and non-motor symptoms. At present, there is no cure for PD. PD is characterized by the progressive loss of dopaminergic (DA) neurons in the substantial nigra (SN) region of the brain and presence of Lewy bodies (LB) consisting mainly of the toxic aggregates of the misfolded α-synuclein in the neurons which are believed cause damage to cellular components. To date, the exact etiology of PD remains unclear.

Recently, dysfunctions of the mitochondria have been found to be a key factor in the DA neuronal deaths in both monogenic and sporadic PD cases. Mitochondria are vital organelles for various cellular functions. They contain the machinery for transcription, translation, and the five protein complexes involved in the oxidative phosphorylation to generate adenosine triphosphate (ATP), the primary source of energy for cellular functions [1]. Defects in the mitochondrial respiratory chain have been described in the homogenized SN lysates obtained from the brain of PD patients. Also, several mitochondrial function associated genes have been associated with increased Parkinson’s risk and late-onset disease [2]. A major bottleneck encountered by the traditional approaches to correct mitochondria-related disorders is the difficulty of targeting drugs or small molecules to specific sub-compartments of the mitochondria. Moreover, the presence of different types of mitochondrial mutations among patients makes it impossible to develop a single drug for this disorder.

Peptide-mediated allogeneic mitochondrial delivery (PMD) has shown benefit for the treatment of PD [3]. However, there are some concerns raised about PMD, such as the purified mitochondria becoming nonfunctional, and the inability of DA neurons to effectively internalize the transplanted mitochondria. Hence, it is imperative to explore new approaches to the delivery of healthy mitochondria into the DA neuron.

The intercellular transfer of mitochondria has been reported both in vivo and in vitro. Multiple shreds of evidence now suggest that it is a physiological process that supports cell survival, rescues aerobic respiration, and protects against external stress in recipient cells [4–7]. For example, recently, it was shown that the transfer of healthy mitochondria to damaged cells represents an important mechanism of endogenous regeneration mediated by the mesenchymal stem cells (MSCs). MSCs can donate mitochondria to cardiomyocytes [8], mouse alveoli [4], lung epithelial cells [9], human and mouse macrophages [10], and cortical neurons [11]. Also, astrocytes have been shown to donate mitochondria to motor neurons after ischemic stroke in a mouse model [12]. The mitochondrial transfer also seems to be involved in stem cell-triggered repair of damaged cells [13]. Based on these studies, it has been proposed that cell stress/injury is required for organelle transfer. Such observations have provided the impetus to investigate the possibility of rescuing DA neurons by intercellular mitochondrial transfer in the PD model.

In this study, we asked whether induced pluripotent stem cell (iPSCs)-derived astrocytes can transfer functional mitochondria to rescue injured DA neurons (iPSC derived) after rotenone exposure in an in vitro model of PD. We chose astrocytes as a cell source for the following reasons. Brain energy metabolism is a complex process in which cooperativities between astrocytes and neurons play a central role. Importantly, the metabolic phenotypes of neurons and astrocytes appear to be largely complementary. The healthy mitochondrial transfer may be essential for these neuroglial protective mechanisms since inhibition of astrocytic mitochondria makes neurons vulnerable to cell death.

In the present study, we showed that spontaneous mitochondrial transfer from astrocytes to DA neurons can rescue neurodegeneration of DA neurons in a PD model of rotenone-induced neurodegeneration. Our studies provided a potential novel cell therapy approach for the treatment of PD.

## Materials & methods

### Stem cell cultures

The human embryonic stem cell (hESCs) line (H9; passage 30–35) was a kind gift from Dr. James Thomson (University of Wisconsin-Madison, Madison, WI, USA), and the iPSC line (UTY-1; passage 33–40) was a kind gift from Dr. Ying Liu (University of Texas Health Science Center at Houston, Houston, USA). Both cell lines were cultured in a feeder-free condition on Matrigel-coated 6 well plates (Corning, USA) with E8 media (Thermo Fisher Scientific, USA). Stem cells were passaged by mechanical cutting every 4 days, and the ROCK inhibitor Y- 27632 (10 μM) was added during the first 24 h after passaging.

### Induction of NSCs

Neural stem cells (NSCs) were derived from undifferentiated hiPSCs/hESCs colonies cultured in 6 well Matrigel-coated plates according to previously published protocols. Neural induction was initiated by exposing the colonies for 5 days to dual SMAD signaling pathway inhibitors 10 μM SB431542 and 500 ng/mL Noggin. The BMP inhibitor (R&D) and transforming growth factor-beta (TGF-βR) inhibitor (Tocris) (Chambers, Fasano, et al. 2009) were added to the basal media consisting of DMEM/F12, NEAA, 2 mM GlutaMax, 1x sodium pyruvate, 1 × 2-Mercaptoethaol, 1x Penicillin-Streptomycin. Upon day 5 of differentiation, the SB431542 was withdrawn and increasing amounts of N2 media (25, 50, 75%) was added to the NSC (DMEM/F12, 1x NEAA, 1x Glutamax, 1x sodium pyruvate, 1 × 2-Mercaptoethaol, 1x Penicillin-Streptomycin) media while maintaining 500 ng/mL of Noggin. NSCs are confirmed by immunohistochemistry of SOX2 and Nestin. NSCs were used or stocked for further differentiation under adherent conditions.

### Differentiation of dopaminergic neurons

As mentioned above neutralization of hiPSCs and hESCs by dual SMAD inhibition permitted a pre-rosette, neural stem cell with dopaminergic and motoneuronal potential on day 5. From day 6 cells were cultured in the dopaminergic neuron induction media (consisting of DMEF/F12, NEAA 1x, GlutaMax 1x, sodium pyruvate 1x, 2-Mercaptoethaol 1x, Penicillin-Streptomycin 1x) with 500 ng/ml Noggin and 200 ng/ml Sonic hedgehog (Shh) (added fresh daily) for 4 days. Cells were then additionally cultured for 3–5 days in the dopaminergic neuron induction media with Brain-Derived Neurotrophic Factor (BDNF, 20 ng/ml, R&D Systems), L-Ascorbic Acid (L-AA, 200 μM, Tocris), Sonic Hedge Hog (SHH, 200 ng/ml, Sigma), and Fibroblast Growth Factor 8b (FGF8b, 100 ng/ml, Sigma). Media was changed on alternate days. This leads to midbrain dopaminergic neuronal patterning within 7–9 days. Immature DA neurons were then further matured by exposure to BDNF (20 ng/ml, R&D Systems), Glial Derived Neurotrophic Factor (GDNF, 20 ng/ml, R&D Systems), Transforming Growth Factor β3 (TGF-β3, 1 μM, R&D Systems), L-AA (200 μM, Tocris) and Cyclic Adenosine Monophosphate (dcAMP, 1 mM, Sigma) in N2 media for 7–11 additional days. DA neurons exhibited typical neuronal morphology including the soma, axons, and dendrites. DA neuronal identity was confirmed through immunocytochemistry of class III beta-tubulin (TUJ1) and Tyrosine hydroxylase (TH) immunoreactivity.

### Astrocyte differentiation

First, iPSCs and hESCs were induced to differentiate into NSCs as described above. Then, the NSCs were plated on a 10 cm dish coated poly-L-ornithine (0.002%) and fibronectin (10 μg/ml, Millipore) in a chemically defined and xeno-free astroglial medium containing DMEM/F12, 1 × N2 supplement, 1 × B27 (without retinoic acid) supplement, bone morphogenic protein 4 (BMP4, 10 ng/ml, Peprotech) and basic fibroblast growth factor (bFGF, 20 ng/ml) for directed astroglial differentiation and maturation for 14 days [14, 15]. Astrocytes were characterized by immunostaining using anti-S100 beta and anti-GFAP antibodies. Cells were fed twice a week and were passaged after they were 80–90% confluent. Cells were passaged at least 5–6 times during astroglial differentiation.

### Immunocytochemistry

DA neurons or astrocytes in cultures were seeded on 20 mm diameter glass coverslips that were individually placed in each well of 12 well plates and were fixed by 4% paraformaldehyde (PFA) for 10 min at room temperature. Cells were then washed and first permeabilized with 0.1% Triton X-100 and blocked in 10% BSA in PBS for 30 min before staining. The cells were incubated overnight at 4 C° with the following primary antibodies: mouse anti-tyrosine hydroxylase (TH) (1:1000, Abcam, ab129991), rabbit anti-class III beta-tubulin (Tuj1) (1:1000, Abcam, ab18207), rabbit anti- glial fibrillary acidic protein (GFAP) (1:2000, Abcam, ab7260), and mouse anti-S100b (11,000, Abcam, ab14849). Then the detection of target antigen was performed by species-appropriate fluorescence-conjugated secondary antibodies Alexa flour 488, 555, or 647. Cells were visualized and imaged by a Zeiss Confocal Microscope.

### Western blotting

Protein levels were assessed by western blotting. DA Neurons or astrocytes were seeded at a density of 0.6 × 10^5^/ cm^2^ or 0.3 × 10^5^/ cm^2^ respectively in 35 mm culture dishes and were harvested for western blotting analysis after they were 90% confluent. Cells were lysed in Lysis buffer consisting of 5 mM EDTA, 150 mM sodium chloride, 25 mM Tris, 1% Nonidet P^− 40^ pH 7.5, with protease inhibitor cocktail mixture (Sigma, St. Louis, MO, USA). We measured the protein concentrations of each sample in the clear supernatants using the Bicinchoninic Acid assay (BCA assay). Proteins with the amount of 30 μg were then loaded onto 10% sodium dodecyl sulfate-polyacrylamide gel electrophoresis (SDS–PAGE) gels. After protein transfer to PVDF membranes, the membranes were blocked in Tris-buffered saline (TBST) with 5% (w/v) milk powder for 60 min at room temperature. The Polyvinylidene difluoride (PVDF) membranes were then incubated with following primary antibodies: anti-GAPDH antibody (1:2000, Sigma-Aldrich, G8795), anti-TH antibody (1:1000, Abcam, ab129991), anti-Tuj1 antibody (1:1000, Abcam, ab18207), anti-GFAP antibody (1:1000, Abcam, ab7260), anti-S100b (1:1000, Abcam, ab14849), anti-phosphorylated p38 (1:1000, Cell Signaling Technology, 4511 T), anti-p38 (1:1000, Cell Signaling Technology, 8690 T), anti-MIRO1 (1:1000, Abcam, ab83779), anti- TNFaIP2 (1:1000, Abcam, ab91235) antibodies, overnight at 4C°. Then the membranes were incubated with horseradish peroxidase (HRP)-conjugated secondary antibodies, and the visualization was enhanced by the ECL chemiluminescence detector (GE Healthcare, NA931 (anti-mouse), NA934 (anti-rabbit), or NA935 (anti-rat)). Optical densities were quantified using the National Institute of Health (NIH) ImageJ software.

### Rotenone induced neurotoxicity

Immature neurons were plated in 12 well plates and cultured in DA neuron maturation media for 7–11 days as mentioned above. At day 22 or 23 neurons were treated with rotenone (100 nM dissolved in 0.1% DMSO which itself showed no toxic effect on the cells at this percentage) added to the culture media for 12 h. Cells were washed with DPSB twice after 12 h that and fresh DA maturation media was added for downstream experiments.

### Establishment of the co-culture System

In one set of experiments, the iPSCs derived astrocytes (6 × 10^4^/cm^2^) were gently placed on top of the iPSCs derived DA neurons (6 × 10^4^/cm^2^ and cultured in DA neurons maturation media for 7–11 days) to establish the co-culture system. The neuron to astrocyte ratio was1:1. In a different setup, astrocytes (6 × 10^4^/insert) were plated on cell culture inserts with 0.4 μm and 1.0 μm aperture filter and then placed into each well of the 12-well plates in which DA neurons (6 × 10^4^/ cm^2^) were plated. Cell culture inserts of pore size 1.0 μm were used to allow the transfer of growth factors, small molecular and most organelle including mitochondria into DA neurons co-culture media. Cell culture inserts of pore size 0.4 μm were used to allow the transfer of growth factors and small molecular but prevent the transfer of mitochondria into the DA neuronal co-culture media. For cell survival and neurite length measurement, 3 random fields (20x objective) were imaged and the neurons were counted and averaged. Three to five independent trials were run.

### Assessment of neuronal damage

We evaluated cell survival by counting the TH positive neurons following different treatments by using the Image J software (NIH, Bethesda, MD, USA). Each merged image was opened in Image J, and manual counting mode was applied by clicking the Multi-point macro add-in. The neurite lengths were measured by Image J. For neurite length analysis, the Tuj1 and TH double-stained images were analyzed by the segmented the line-model tool. The length of each curved neurite was measured as short straight segments. The sum of the values of each neurite was determined by adding all the segments. Three random fields (20x objective) were imaged and analyzes. Three independent trials were run.

### Mitochondrial tracking

iPSC derived astrocytes were incubated with the Mito-Tracker probes (Invitrogen/Molecular Probes, USA, 100 nM) for 30 min (according to the manufacturer’s instructions) which results in passive diffusion of the dye through the cell membrane and accumulation in active mitochondria. Once the astrocyte mitochondria are labeled with Mito-tracker, their movement and translocation into other cells can be detected by confocal fluorescence microscopy.

### Flow Cytometry

Flow cytometric analysis was performed by the Thermo-fisher Attune NxT flow cytometer. Cells were collected from the co-culture system, in which the astrocytes were labeled with the Mito-Tracker Green, and DA neurons were labeled with CellTrace Red. First, the cell dissociation enzyme Accutase was used to detach the adherent cells according to the manufacturer’s instructions. Cells were then washed once with Dulbecco’s phosphate-buffered saline (DPBS) and fixed by 4% paraformaldehyde (in PFA) for 10 min. Astrocyte-conditioned media (ACM) was collected from iPSC derived astrocyte cultures after 24 h after the addition of fresh media. The supernatants were filtered through a 0.22-μm syringe filter to obtain mitochondria depleted ACM (dmACM).

### Cell viability assays

The viability of the DA neurons was assessed by using the Cell Counting Kit-8 (CCK-8, Sigma-Aldrich) according to the manufacturer’s instructions. Briefly, an appropriate volume of CCK-8 solution was added into each well of the 12 or 96 well plates and incubated in a BOD incubator for 2 h. A microplate reader was then used to measure the absorbance at 450 nm.

For the co-culture system, DA neurons were plated onto 12-well plates precoated with laminin, and iPSC derived astrocytes were plated on to the Millicell cell culture inserts. The inserts were of 12 mm diameter and fitted with 0.4 μm or 1.0 μm pore size filters respectively. CCK8 (10% of the culture medium volume) was added after the cell culture inserts were removed, and the absorbance was determined at 450 nm. DA neurons that were cultured in only the ACM or dmACM were seeded into 96-well plates, and 10 μL CCK-8 solution was used to conduct the cell viability assay. The values obtained from the same number of control DA neurons plated and cultured in either 12 or 96 well plates were considered as values for 100% survival. Assays were run in triplicates.

### ATP measurement

Cell Titer-Glo luminescence kit (Promega, G7570) was employed to determine the levels of intracellular or extracellular ATP according to the manufacturer’s instructions. This kit can perform cell lysis and generate a luminescent signal proportional to the amount of ATP present. Typically, DA neurons were seeded into 12-well plates (70–80% confluent), while astrocytes were plated into cell culture insert (70–80% confluent) with 0.4 μm or 1.0 μm pore polycarbonate membrane respectively. After 24 h co-culture, the transwell plates were removed and the lyophilized enzyme-substrate mixtures were added into the neuronal culture system and incubated for 30 min at room temperature. Then equal aliquots (50 μl) of the cell lysate or control media were transferred into an opaque-walled 96-well plate, and the luminescence (representing the amount of ATP) was recorded by a luminescence microplate reader. The background luminescence was obtained by the measurement on the control neuronal medium without any neurons.

### Mitochondrial membrane potential measurement

Astrocytes were plated onto a100mm culture dish at a density of 0.6 × 10^5^/cm^2^ in astrocyte media. After 24 h the astrocytic conditioned media (ACM) was collected, and the mitochondria depleted ACM (dmACM) was obtained by passing the ACM through the 0.22 μm sterile filter. The mitochondrial membrane potentials were measured by the JC-1 mitochondrial potential sensors (Thermo Fisher Scientific, USA) according to the manufacturer’s instructions. JC-1 is a cationic dye that exhibits potential-dependent accumulation in mitochondria, indicated by a fluorescence emission shift from green (~ 525 nm) to red (~ 590 nm). Consequently, mitochondrial depolarization is indicated by a decrease in the red/green fluorescence intensity ratio. These characteristics make JC-1 a sensitive marker for mitochondrial membrane potential. JC-1 dye (5 μM, 2 μg/ml) was added to ACM or dmACM and incubated for 30 min at room temperature before assaying. Mitochondria membrane potential was determined by the fluorescent ratio with a fluorescent microplate reader.

### Oxygen consumption analysis

MITO-ID extracellular O_2_ sensor kit (ENZ-51045, Enzo Life Science, USA) was used to measure the real-time oxygen consumption in the ACM or dmACM, as well as in the DA neurons according to the manufacturer’s instructions. This kit contains a phosphorescent oxygen-sensitive reagent enabling high-throughput and real-time oxygen consumption readout. In this assay, MITO-ID® Extracellular O2 SensorProbe is quenched by oxygen, through molecular collision, and thus the amount of fluorescence signal is inversely proportional to the amount of extracellular oxygen in the sample. Rates of oxygen consumption are calculated from the changes in fluorescence signal over time. The reaction is the non-destructive and fully reversible facilitating measurement of time courses. The addition of high-sensitivity mineral oil (supplied with the kit) is used to limit the back diffusion of ambient oxygen. Briefly, DA neurons, ACM and dmACM were prepared as previously described. The MITO-ID extracellular O_2_ sensor probe was reconstituted in PBS according to manufacturer’s instructions, and 1/15 of the medium volume of the reconstituted probe was added to 150 μl of ACM or dmACM or neurons. The plate was read with a filter combination of 340 nm for excitation and 642 nm of emission at 30 °C at different time points after adding two drops (90 μl) of pre-warmed Mito-ID mineral oil.

### Transient transfection with siRNA

For transient transfection, the control siRNA and Miro1 siRNA (GenePharma, Shanghai, China) were used. The target sequences were as follows: human Miro1 siRNA, 5′-UGUGGAGUGUUCAGCGAAA-3′ and 5′-GCAAUUAGCAGAGGCGUUA-3′. Human siRNA non-targeting, 5′-UGGUUUACAUGUCGACUAA-3′ was used. For transient transfection, neurons were transfected with siRNA using Lipofectamine 3000 transfection reagent (Invitrogen, USA) according to the manufacturer’s instructions. Western blotting was applied to assess the efficiency of target gene expression knockdown at the protein level after 24 h post-transfection.

### Statistical analysis

All data are expressed as mean ± SEM. When only two experimental groups were compared, the unpaired t-test was used. Multiple comparisons were treated by one-way ANOVA followed by Tukey’s test. Prism 5 software (GraphPad) was performed for Statistical analyses. *P* values of < 0.05 were considered statistically significant.

## Results

### Generation of DA neurons and astrocytes from hiPSCs and hESCs

**Fig. 1:**
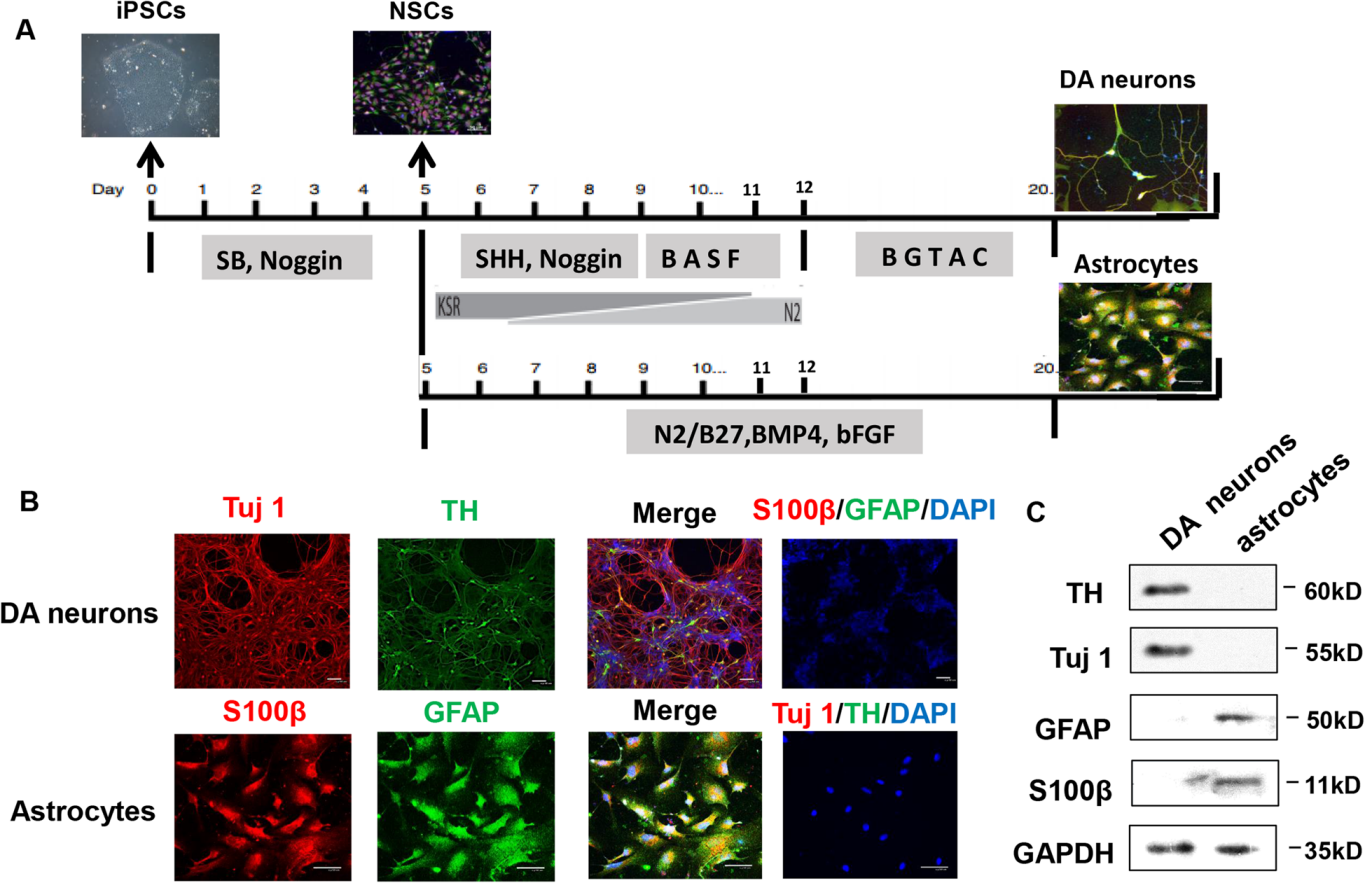
Generation of iPSCs-derived DA neurons and astrocytes. **a** The protocol for the generation of DA neurons and astrocytes from iPSCs. **b** Immunocytochemistry of DA neuron-specific markers anti-TH (green) and anti-Tuj1 (red), as well as astrocytes-specific markers anti-GFAP (green) and anti-S100β (red). **c** Representative immunoblots for TH and Tuj1 expression in DA neurons, and GFAP and S100β expression in astrocytes. Three independent experiments were performed (*N* = 3). Scale bars in all panels represent 50 μm

Previous studies have demonstrated that hESCs and hiPSCs can be efficiently differentiated into functional DA neurons [16, 17] and astrocytes. In this study, we initially followed previously published protocols with slight modifications to derive DA neurons and astrocytes from hiPSCs and hESCs (Fig. 1a). The mature DA neurons were generated from the neuronal stem cells (NSCs) by first culturing them in DA neuron induction media and subsequently in DA neuron maturation media for a total of around 20 days. NSCs are identified by immunohistochemistry of SOX2 and Nestin (Extended Fig. 1). The neurons were positive for the neuronal marker Tuj1 and for dopaminergic neuronal marker tyrosine hydroxylase (TH) (Fig. 1b). The presence of Tuj1 and TH proteins was verified by western blot (Fig. 1c). For astrocytes differentiation, matured astrocytes were obtained from NSCs by 14 days of additional culture in the presence of BMP4. Astrocytes gained a flat star-shaped morphology at the end of this period. Astrocytes were positive for anti-GFAP and anti-S100β immune markers (Fig. 1b, lower panel), and the expressions of GFAP and S100β proteins were verified in western blots (Fig. 1c). We found similar efficiency of generation of DA neurons and astrocytes from both cell line, hence all subsequent experiments were done using iPSC derived neurons and astrocytes.

### Astrocytes prevented rotenone-induced DA neurodegeneration in a co-culture system

**Fig. 2:**
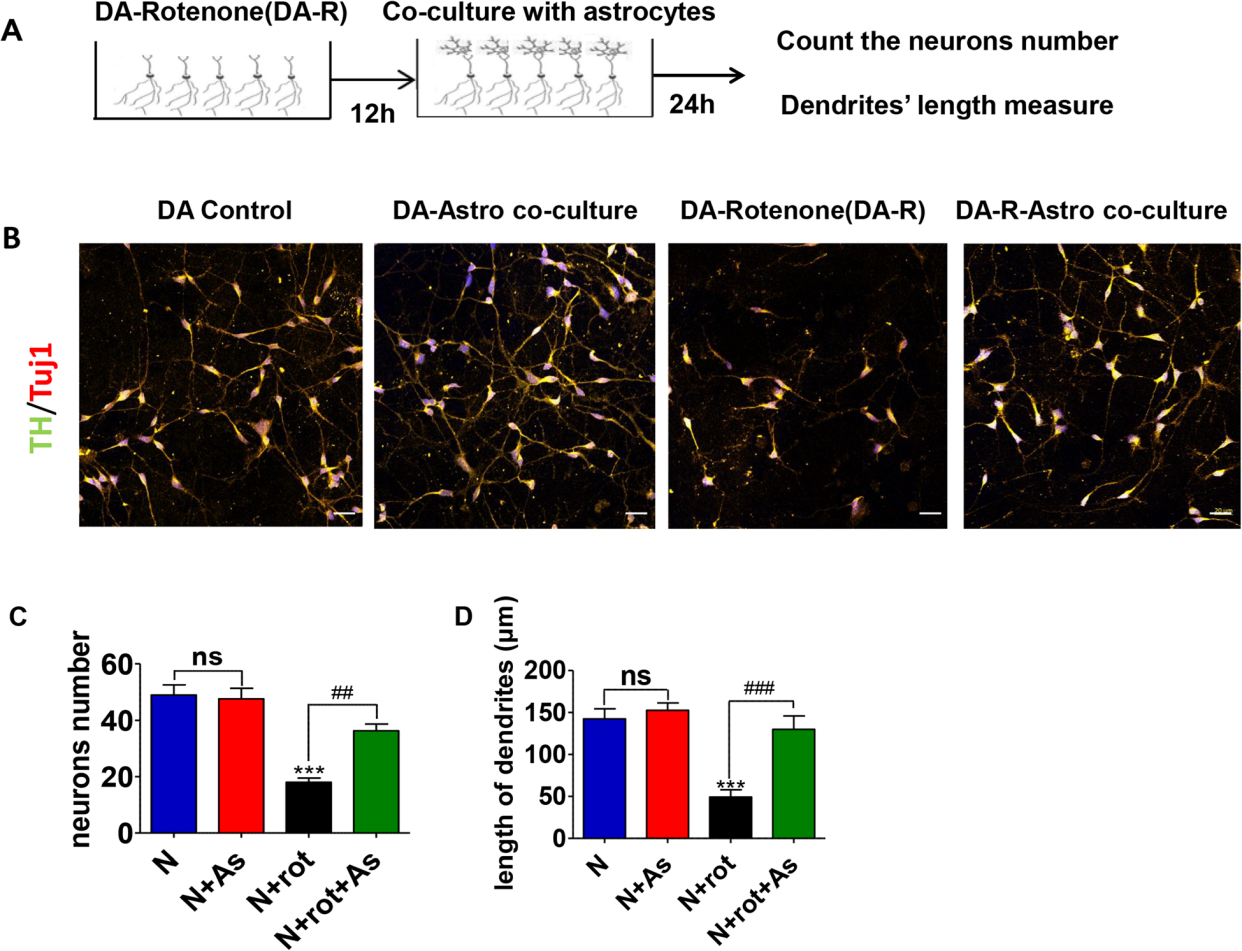
Reversal of rotenone-induced injuries in DA neurons after direct co-culture with iPSCs derived astrocytes. **a** The schematics of the astrocyte-neuron co-culture experiments. **b** The representative images of DA neurons in four experiment groups: healthy control group, co-culture group, rotenone-treated group, co-culture after rotenone exposure group. **c** Quantification of TH and Tuj1 positive cell numbers in those four groups. **d** Quantification of neurite lengths of the DA neurons (TH and Tuj1 double staining). Three slides from each group were quantified and averaged 5 independent experiments. Results were presented as mean ± SEM. *** *P* < 0.001 versus controls, ^##^*P* < 0.01, and ^###^*P* < 0.001 compared to rotenone. Scale bars in all panels represent 20 μm

iPSCs-derived DA neurons were exposed to 100 nM rotenone, a mitochondrial complex I inhibitor, for 12 h. Astrocytes were then added on to the DA neuronal cultures and co-cultured for 24 h (schematics shown in Fig. 2a). Similar volumes of extra media were added to controls rotenone treated DA neurons. Health DA neurons co-cultured with astrocytes did not show any change in the number and neurite length. Rotenone exposure lead to death ~ 64% of TH-positive neurons (average neuronal count in DMSO control was 49.0 ± 3.6 verses rotenone 18.0 ± 1.5, *P* < 0.001) (Fig. 2b and c), and 80% reduction the length of the TH positive neurites compared to DMSO-control DA neurons (control 142.5 ± 11.9 μm vs. rotenone 49.3 ± 8.6 μm, *P* < 0.001) (Fig. 2b and d) after 12 h exposure. Co-cultures with astrocytes significantly reversed rotenone-induced TH neuron loss (average neuronal count in rotenone was 18.0 ± 1.5 vs. rotenone + astrocyte 36.3 ± 2.4, *P* < 0.05) and their reduced neurite length (average length of neurites in rotenone was 49.3 + 8.6 μm vs. rotenone + astrocyte 128.9 + 16.1 μm, *P* < 0.001) compared to rotenone group (Fig. 2b-d).

### iPSCs-derived astrocytes could act as a mitochondrial donor to rotenone injured DA neurons

**Fig. 3:**
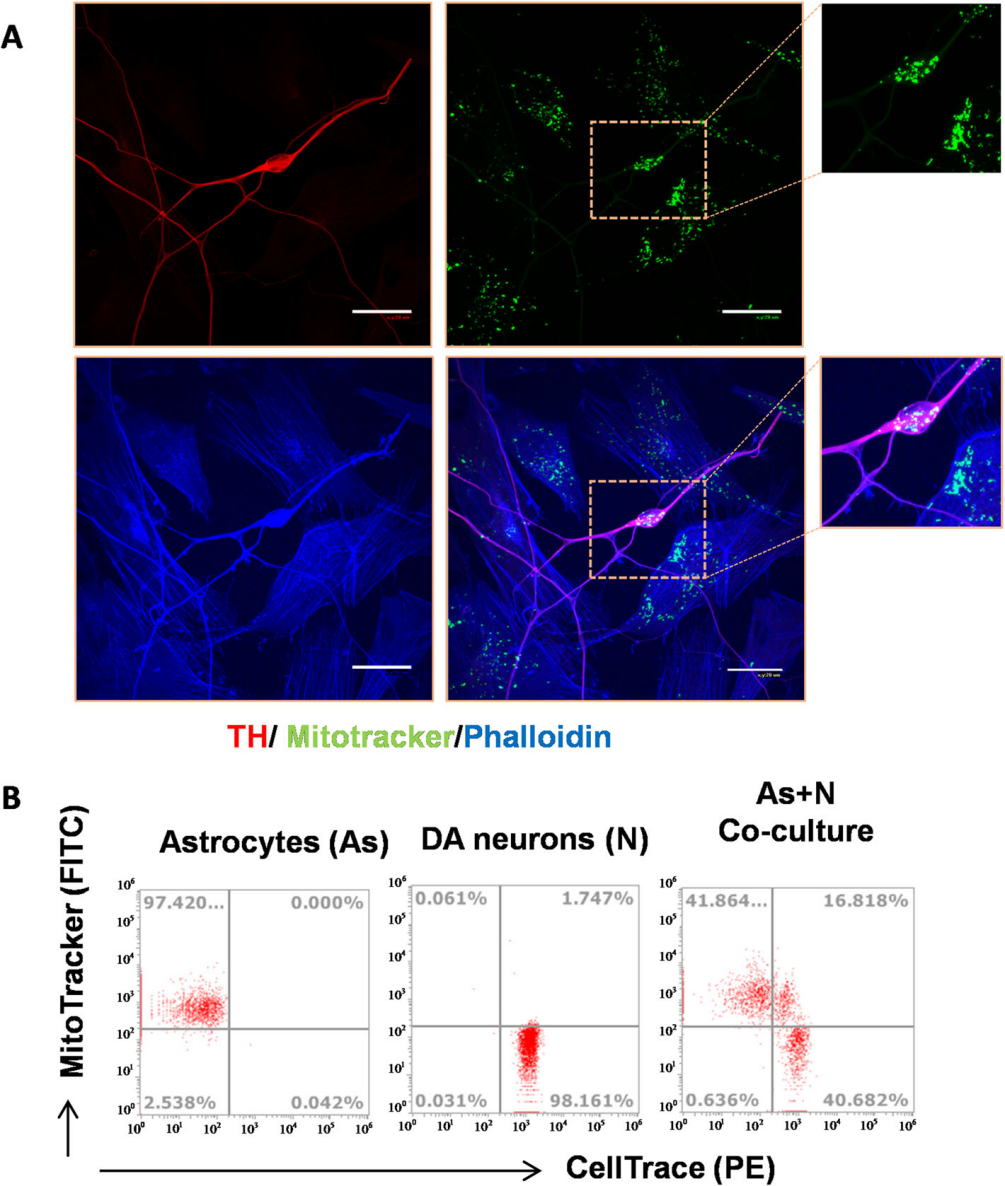
Astrocytic originated mitochondria transfer to injured DA neurons in a rotenone-induced PD in vitro model. **a** Mitochondria in astrocytes were labeled with Mito-tracker (green) before co-culture with DA neurons. 24 h after co-culture, immunostaining showed that astrocytic mitochondria were present in DA neurons. Phalloidin dye (blue) and TH (red) were employed to outline the astrocytes and DA neurons respectively. **b** The presence of astrocytic mitochondria in CellTrace labeled DA neurons was analyzed by flow cytometry. Astrocytic mitochondria were stained with Mitotracker green separately and neurons were labeled with CellTrace (violet) before co-culture. The efficient transfer of Mitochondria Tracker Green labeled mitochondria was confirmed in co-cultures by the presence of dual-positive cells is composed of Mitotracker and CellTrace analyzed by flow cytometry. Three independent experiments (*N* = 3). Scale bars in all panels represent 20 μm

To investigate if a mitochondrial transfer was involved in the iPSCs derived astrocytes mediated neuroprotection in DA neurons, the astrocytic mitochondria were labeled with the dye Mito-Tracker Green, and then were added to the rotenone pre-treated DA neuronal cultures and co-cultured for 24 h. The tagged mitochondria were tracked under a confocal fluorescence microscope. Cells were then immunostained with anti-TH antibody (DA neuronal marker) along with the phalloidin dye for the filamentous actin (F-actin). We observed a significant number of Mito-Tracker Green labeled mitochondria inside the TH positive neurons (Fig. 3a). These results indicated that labeled mitochondria in the neurons originated from astrocytes. We further validated these results by flow cytometry to detect astrocytic mitochondria in DA neurons (Fig. 3b).

### The neuroprotective effects of the astrocytes were not due to growth factor release, but mostly from mitochondrial transfer to DA neurons

**Fig. 4:**
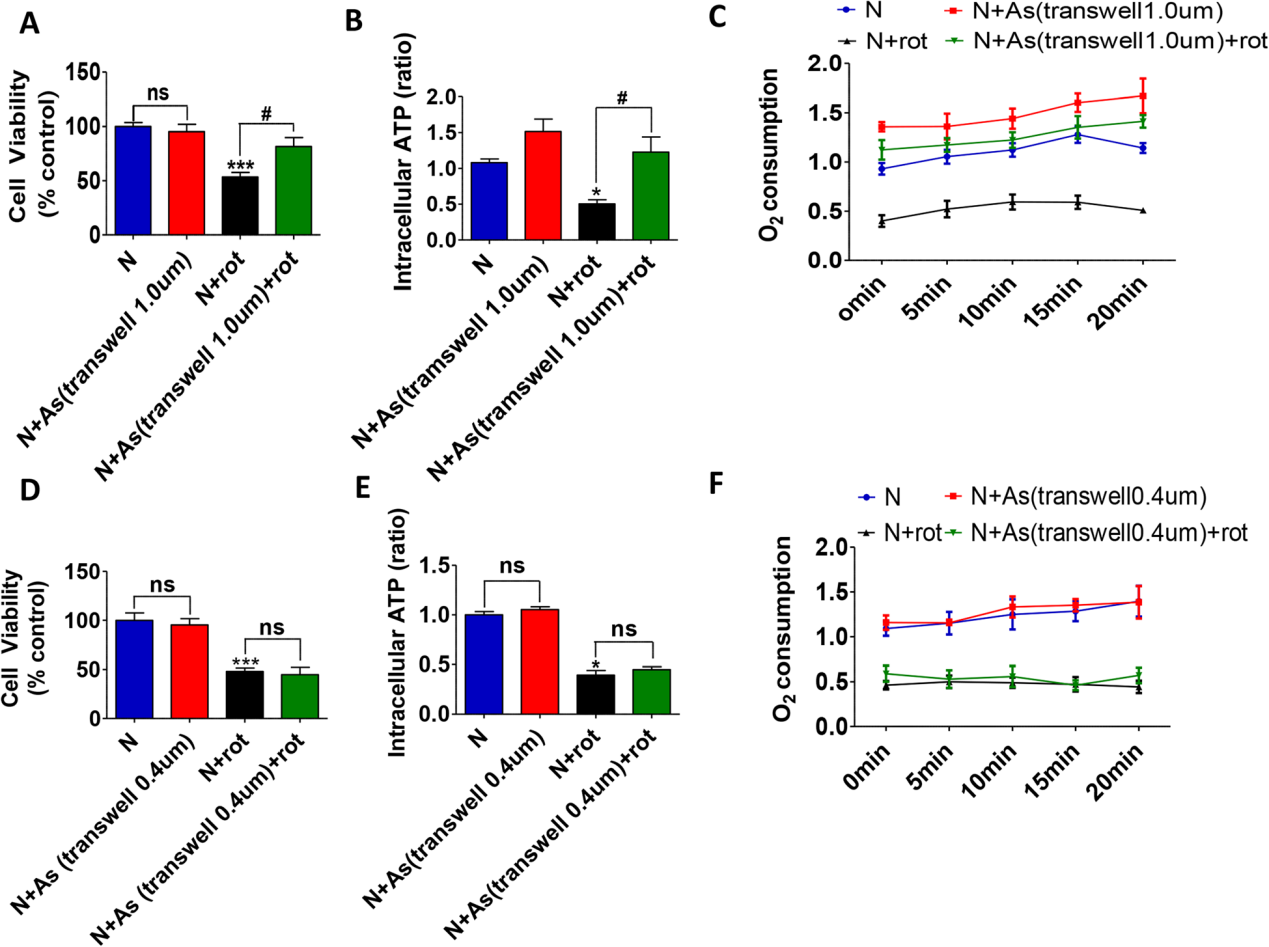
The reversal of rotenone-induced DA neuron toxicity is associated with the mitochondria transfer from astrocytes to DA neurons. **a-b** Astrocyte was plated on cell culture inserts (fitted with 1.0 μM or 0.4 μm filters) for co-cultures to either allow or prevent access of astrocyte-derived mitochondria to DA neurons (The filtration membranes of pore size 1.0 μm allowed whereas 0.4 μm excluded the released mitochondria to pass into the co-culture media). Co-cultures with 1.0 μm filters increased the cell viability of DA neurons following rotenone (**a**), while the protective role was eliminated when the mitochondria were prevented to go into the media by the 0.4 μm filters (**d**). **b, e** The intracellular ATP of DA neurons showed similar trends. The intraneuronal ATP levels fell significantly (~ 50%) after rotenone (N + rot), which were significantly elevated after co-culture with astrocytes seeded on cell culture insert with 1.0 μm filters (**b**). In contrast, there was no recovery of intraneuronal ATP levels in the co-culture system containing a cell culture insert with a 0.4 μm filter (**e**). **c, f** Similar changes were observed with the real-time oxygen consumption rate (OCR) analysis. The OCR in rotenone-neurons was decreased, indicating a reduction in neuronal respiration and mitochondrial function, which was recovered in the co-culture system containing the 1.0 μm filters allowing mitochondria transfer. Results were presented as mean ± SEM of the triplicate assay in each experiment. Three independent experiments (*N* = 3). * *P* < 0.05, *** *P* < 0.001, and ^#^*P* < 0.05

We then investigated whether the neuronal protection was due to mitochondrial transfer or it was associated with the paracrine action of astrocytes. Astrocytes were grown on cell culture inserts with filtration membranes of pore sizes of 0.4 μm and 1.0 μm respectively. Mitochondria from astrocytes can usually pass through 1.0 μm diameter aperture filters while it would be prevented by a 0.4 μm diameter aperture filter. Under such conditions, nutrient and growth factors secreted from astrocytes could communicate with neuronal medium freely. Cell viability reduced by 50% after rotenone treatment (N 100.0 ± 0.0 verses N + rot 53.4 ± 4.2%, *P* < 0.001). The viability of rotenone treated DA neurons were 81.5 ± 8.2% of controls when they were co-cultured with astrocytes grown on transwell containing 1.0 μm filters (Fig. 4a), which showed a significant protective effect. However, the neuroprotective effects were abolished in co-cultures with astrocytes plated on transwell containing 0.4 μm filters (Fig. 4d). These results excluded the possibility that the astrocytic paracrine action contributed to the neuroprotective effects.

### Transferred mitochondria from astrocytes restored the rotenone-induced ATP depletion in DA neurons

We measured the intracellular levels of ATP and real-time oxygen consumption rate (OCR) in the control DA neurons, after exposure to rotenone and in rotenone treated neurons subjected to co-cultures with astrocytes grown on inserts. The interneuronal levels of ATP measured 24 h after the end of 12 h’ rotenone exposure was found to be reduced by around 50% compared to controls in both setups (*P* < 0.05) (Fig. 4b and e respectively). When the control DA neurons were co-cultured with astrocytes plated on 1.0 μM filters containing transwells, the levels of neuronal ATP were elevated (by ~ 43%) but did not reach statistical significance (Fig. 4b), indicating that some degree of mitochondria transfer may take place under healthy conditions too. In rotenone exposed groups, the ATP levels were significantly (P < 0.05) restored in the neurons only when the transwell inserts had a pore diameter of 1.0 μm (Fig. 4b), while no restoration of neuronal ATP levels was seen in the 0.4 μm filter co-culture system (Fig. 4e). The cellular OCR analysis was also employed to determine the respiratory function of DA neurons, as a supplement to ATP. The real-time OCR showed a similar pattern of changes as ATP. Rotenone exposure leads to a decrease in oxygen consumption by the neurons, suggesting reversible damage of neuronal respiration since the rate of oxygen consumption was recovered in the 1.0 μm filter co-culture system (Fig. 4c). However, the 0.4 μm filter co-culture system did not reverse the reduction in oxygen consumption (Fig. 4f).

### Mitochondria were transferred to the neurons via uptake from the media and not via tunneling nanotubes

The F-actin filaments participate in the formation of tunneling nanotube (TNT) between cells for intercellular organelle exchange, including mitochondrial. To investigate the mechanism of mitochondrial transfer from astrocytes to neurons, co-cultures were stained with the F-actin probe phalloidin and subjected to confocal laser microscopy for detection of TNT structure. However, we did not observe the formation of any TNT-like structure between the DA neurons and astrocytes. However, thin membranous TNT like structures was seen between astrocytes (Extended Fig. 2). Astrocytes have been reported to show the reorganization of the actin network and form the TNT structure under oxidative stress [18, 19].

### Astrocyte derived mitochondria released in the ACM were functional

**Fig. 5:**
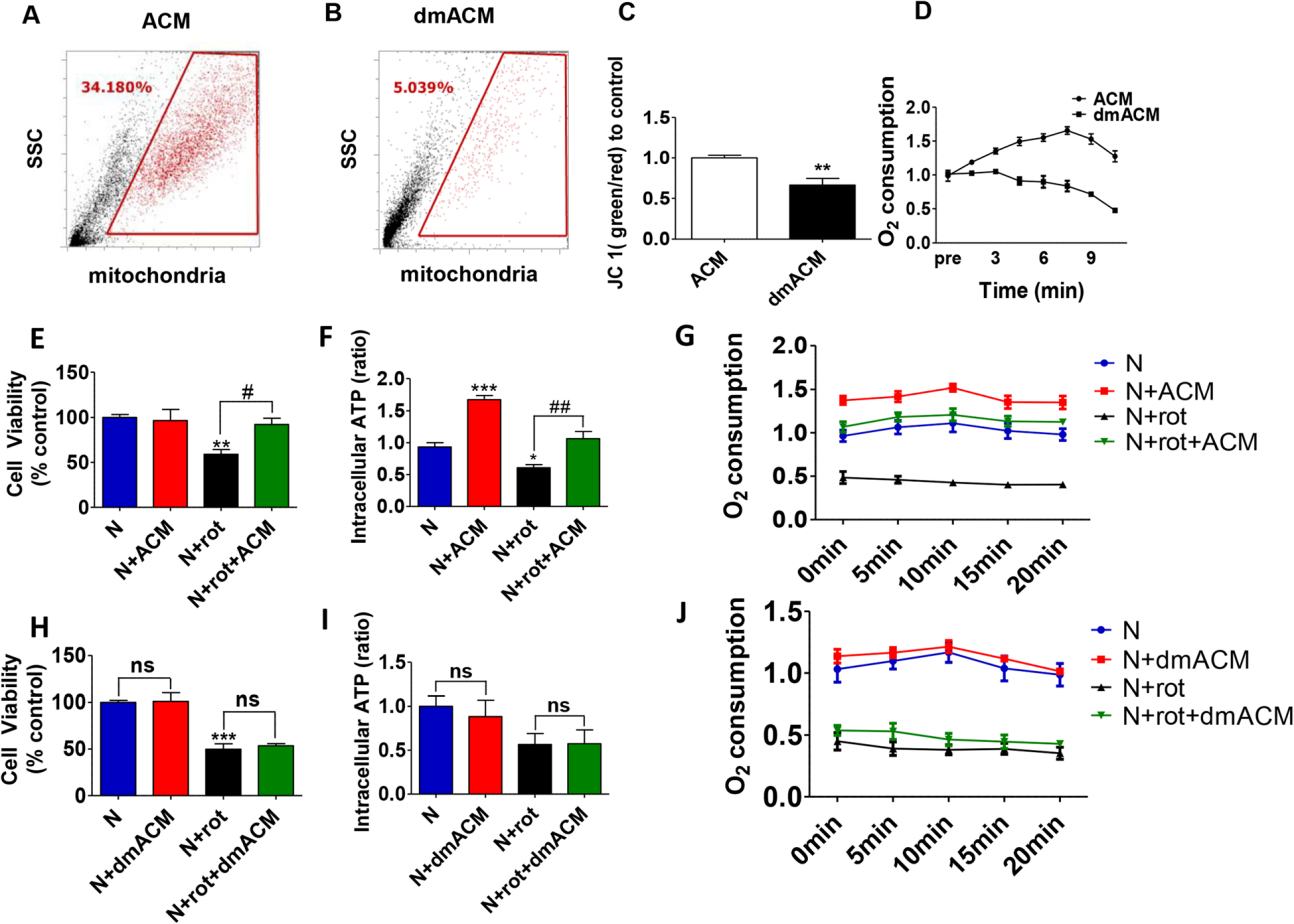
Astrocyte-derived mitochondria released into the medium were functional and neuroprotective. **a**-**b** iPSCs derived astrocytes were labeled with Mito-Tracker Green. Representative flow cytometry data plots (gating optimized for mitochondrial size adjustments) show the presence of astrocytic mitochondria in the media (ACM 34.1%) (**a**), while filtration through 0.22 μm filters depleted mitochondria in the ACM (dmACM 5.0%) (**b**). **c**-**d** The depletion in mitochondria in dmACM was indicated by a reduction in mitochondrial membrane potential (JC1 ratio) and a real-time O_2_ consumption rate (OCR). **e**-**f** The intraneuronal ATP levels and cellular viabilities increased significantly when the DA neurons were cultured with control ACM after the treatment of rotenone. **g** Similar changes were observed by the OCR analysis. **h**-**j** The intraneuronal ATP levels and reduced cellular viabilities could not reverse in dmACM, as well as the real-time OCR records. *N* = 3, results were presented as mean + SEM. * *P* < 0.05, ** *P* < 0.01, *** *P* < 0.001, ^#^*P* < 0.05, and ^##^*P* < 0.01

To assess whether the astrocytes released intact and functional mitochondria into the ACM directly, we used flow cytometry to detect mitochondrial particles in ACM. After size detection and gating for intact mitochondria, we detected a significant percentage (~ 34.1%, *P* < 0.001) of Mito-Tracker Green labeled mitochondria in the ACM, in contrast only 5% mitochondrial particle was detected in mitochondria depleted ACM (dmACM) (Fig. 5a and b respectively). The comparison of the mitochondrial membrane potentials and oxygen consumption between ACM and dmACM demonstrated that these Mito-tracker labeled mitochondria were functional (Fig. 5c-d). Consequently, when the ACM was passed through a 0.22 μm filters to remove mitochondria (dmACM), the recorded mitochondrial membrane potential (Fig. 5c) and oxygen consumption values (Fig. 5d) fell significantly. The ACM could rescue rotenone-induced DA neuron degeneration (Fig. 5e), similar to the results seen with the direct astrocytes-neuron co-culture system. Conversely, culturing the neurons in the dmACM did not reverse the rotenone-induced toxicity (Fig. 5h). These data suggested that cell to cell contact was not a prerequisite for astrocyte mediated neuroprotection and mitochondrial transfer. After rotenone exposure, there was a significant reduction (*P* < 0.05 vs control DA neurons) in the ATP levels and OCR which recovered back to the control DA neuronal levels in the presence of ACM (Fig. 5f, g). The changes in the uptake of mitochondria by DA neurons were quantified by flow cytometric analysis. In the healthy DA neuron group, neuronal uptake was around 22% of total mitochondria. However, when the DA neurons were treated with rotenone, the mitochondria uptake ratio increased to ~ 39% (Extended Fig. 3a, b), indicating that rotenone exposure enhanced the mitochondria uptake. On the contrary, when healthy DA neurons were cultured in the presence of dmACM, there was no change in the ATP levels and OCR in the neurons. In addition, dm ACM failed to recover the neuronal ATP levels and OCR after rotenone exposure (Fig. 5i, j).

### p38 MAPK signal pathway mediated DA neurons uptake of astrocyte-derived mitochondria

**Fig. 6:**
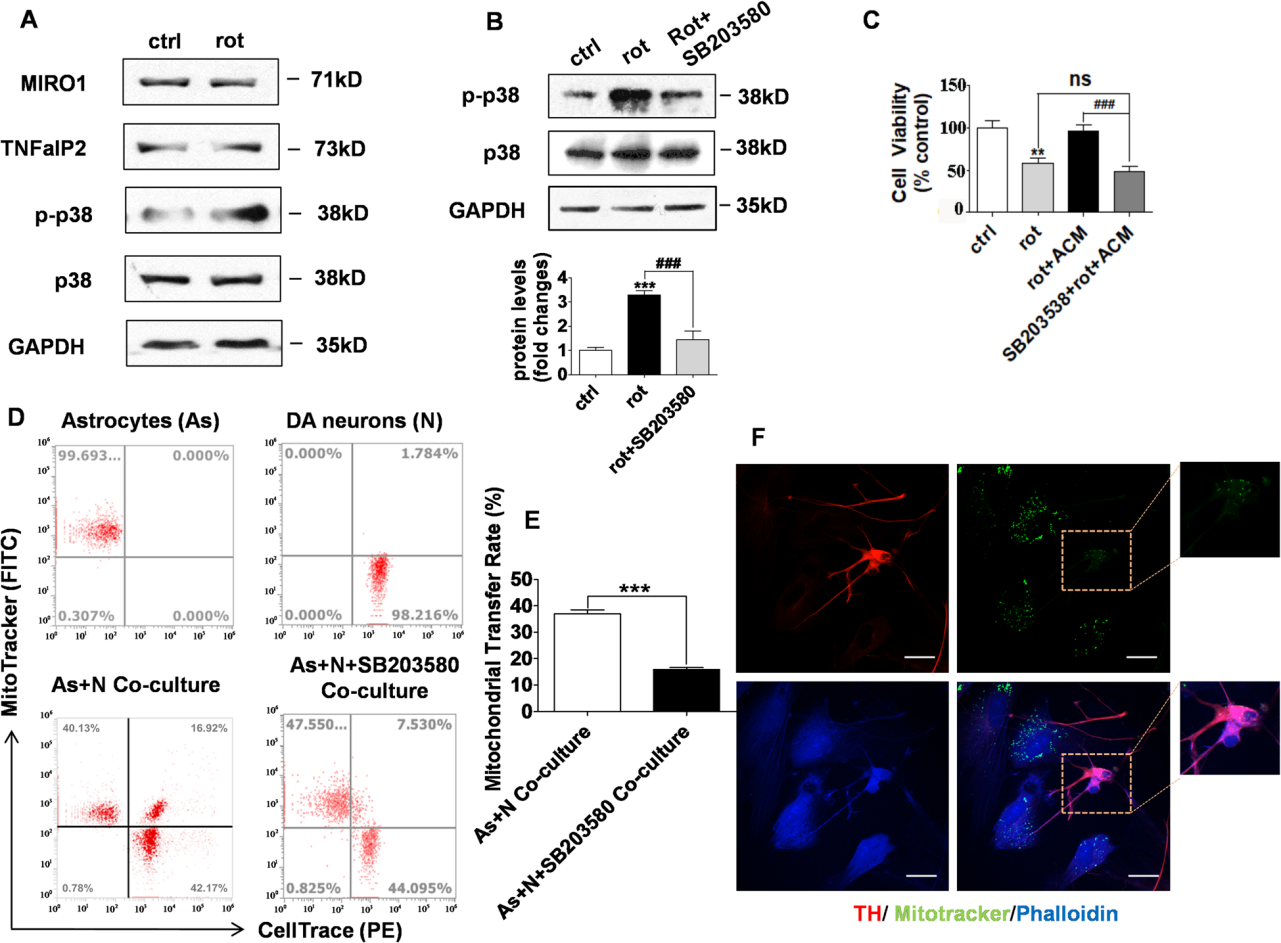
The Phosphorylation of p38 mediated the neuronal uptake of astrocytic mitochondria. **a** Representative Western blot plots showing protein levels of MRIO1, TNFaIP2, total and phosphorylated-p38 (p-P38) and GAPDH in control DA neurons and after rotenone exposure. **b** An inhibitor of p-38, SB203580, inhibited the rotenone-induced increase in the p-p38 protein levels in neurons. **c** The neuroprotective role of ACM was abolished by the pre-treatment with SB203580 which inhibits p38 mitogen-activated protein kinase phosphorylation. **d** Flow cytometry data plot shows the percentage of recipient DA neurons that took up mitochondria from astrocytes with or without SB203580 exposure. The first plot (top left) shows MitoTracker Green labeled donor astrocytes in the first quadrant. The second plot (top right) shows CellTrace Red labeled recipient DA neurons in the third quadrant. The third plot (bottom left) shows in the co-culture setup in which the double-positive (green and red) DA neurons are in the second quadrant. The fourth plot (bottom right) also shows the double-positive DA neurons in the second quadrant. The uptake of mitochondria was decreased when neurons were pre-treated with SB203580, and the mitochondrial transfer rate was reduced from 29.3% ± 1.3% to 14.6% ± 0.6% (**e**). **f** Immunostaining was done to detect astrocytic derived mitochondria in TH labeled (red) DA neurons. *N* = 3, results were presented as mean + SEM. ** *P* < 0.01, *** *P* < 0.001 and ^###^*P* < 0.001 versus controls. Scale bars in all panels represent 20 μm

To explore the mechanism of the neuronal uptake of mitochondria from the ACM, we assessed the roles of three key signaling pathways that may be involved in mitochondrial or vesicle traffic including the Rho GTPase 1 (MIRO1) pathway, TNF-a/NF-kB/TNFaIP2 signaling pathway, and p38 MAPK pathway. By western blot analysis, we did not find any change in the Miro1 and TNFaIP2 proteins in neurons after rotenone treatment. However, the levels of the phosphorylated-p38 (p-p38) were significantly elevated in rotenone-treated DA neurons, while p38 levels remained unchanged (Fig. 6a). SB203580 is an inhibitor of p-38, was used to reduce the rotenone-induced p-p38 levels (Fig. 6b). Treatment with 10 μM SB203580, an inhibitor of p38 MAPK signal pathway suppressed p38 phosphorylation and abolished the neuroprotection from ACM against rotenone-induced neurotoxicity (Fig. 6c). Moreover, SB203580 also reduced the astrocytic mitochondria transfer to neurons, as determined by flow cytometry and immunostaining assays (Fig. 6d-e). However, considering that Miro 1 regulates mitochondrial trafficking, and its activation can play a role in mitochondrial uptake, we assessed the mitochondrial transfer by flow cytometry following Miro 1 knock-down by transfection of Miro1 siRNA in DA neurons. The neurons which had accumulated Mito-Tracker Green positive astrocytic mitochondria accounted for ~ 19% of total cells (see extended Fig. 4a, b)., indicating similar shuttle efficiency seen without the knockdown of Miro 1 (see Fig. 3b). That at least in this model Miro1 was not involved in the regulation of mitochondrial shuttle.

## Discussion

In this study, the major finding is that human iPSCs-derived astrocytes can act as mitochondrial donors supplying healthy mitochondria to rescue rotenone-induced DA neuronal degeneration in vitro. To our knowledge, this is the first report that, like primary astrocytes, iPSCs-derived astrocytes release functional mitochondria in the extracellular media that can be taken up by DA neurons via a p38 signaling pathway.

In this study, we first generate astrocytes and DA neurons from iPSCs by established protocols and characterized them by the expressions of S100β/GFAP and Tuj1/TH respectively. We also used rotenone as a neurotoxic agent to induce DA neuron degeneration to model the PD pathology in vitro [3, 20, 21].

We found that 12-h rotenone exposure led to marked pruning of dendritic structures (marked by loss of TH positive processes) and death of significant numbers DA neurons compared to the control group. Rotenone is a pesticide and a potent mitochondrial complex I inhibitor. It is known to cause neuronal toxicity [22] via multiple pathophysiological mechanisms. Rotenone has been strongly linked to pathophysiological mechanisms implicated in experimental models of human Parkinson’s disease [23].

When the rotenone treated DA neurons were subsequently cultured in the presence of autologous iPSCs-derived astrocytes, the dendrite lengths were maintained and there was significantly less neuronal death, indicating that these astrocytes could reduce the effect of rotenone-against these degenerative changes. In order to follow the movement of mitochondria, we had tagged the astrocytic mitochondria using Mito-Tracker Green. Using immunostaining and flow cytometry we could detect astrocytic-derived mitochondria (Mito-Tracker green positive) within the soma and axons of DA neurons that were co-cultured with astrocytes, suggesting that the mitochondrial transfer indeed took place from astrocytes to neurons. This indicated the possibility that some of the neuroprotective effects may have been via mitochondrial replacements. However, a possibility existed that astrocyte-derived neurotrophic factors released into the ACM could have rescued the neurons. In order to investigate the role of growth factors, a cell culture inserts based co-culture system was applied to allow or prevent mitochondrial access to DA neuronal culture media. We found that the co-culture (containing growth factors) did not rescue the neuronal death and neurite damage when the astrocytes were plated on the 0.4 μm diameter aperture filter. This observation led us to believe that a major part of the neuroprotection from astrocytes came from mitochondrial transfer rather than any other paracrine action.

Next, we sought to identify the cellular uptake mechanism for mitochondria by the DA neurons. It is now apparent that cell stress or injury associated signals may act as a trigger for organelle exchange. TNT is a cytoskeletal-derive structure consistent with F-actin, which allows the intercellular organelle to communicate between cells. The cytoplasmic bridge structure termed TNT has been seen in serval co-culture models [9, 24, 25]. However, we didn’t find any TNTs like structure between astrocytes and DA neurons. The process of exocytosis and endocytosis of mitochondria to and from the extracellular space would be an alternative strategy for uptake by neurons. In support of our theory, we detected the presence of a robust number of free mitochondria in ACM by flow cytometry with Mito-Tracker Green staining and confirmed that they were functional extracellular mitochondrial particles by JC 1 staining and real-time oxygen consumption assays. In addition, consistent with the neuroprotective effect of astrocytes, ACM could rescue the rotenone-treated neurons and restore the respiratory function. In contrast, mitochondria depleted (dmACM) was unable to reverse the neuronal damage. These data together with the earlier data indicated that functional mitochondria from astrocytes were released into the medium.

Several pathways have been suggested to be involved in the release of mitochondria into the extracellular space. hESCs derived astrocytes and mouse primary astrocytes have been shown to release mitochondria in the extracellular media by a calcium depended mechanism. The activation of the CD38-cADPR-calcium signaling pathway facilitates the mouse astrocytic release of functional mitochondria [12]. Mitochondrial Rho GTPase 1(Miro1) is one of the most important proteins for mitochondrial movement, which is located on the outer membrane of mitochondria and is associated with other proteins such as Trafficking Kinesin Protein 1 (TRAK1) and Trafficking Kinesin Protein 2 (TRAK2) for mitochondrial transportation. Tumor necrosis factor-alpha (TNF)-a/neurofilament-kappa B) NF-kB/ TNF Alpha Induced Protein 2 (TNFaIP2) signaling pathway is another mediator of mitochondrial translocation [8]. The p38 MAP kinase-regulated endocytosis is mediated by Clathrin or Rab5, the complex depended on endocytic trafficking [26]. We measured the level of Miro1and TNFaIP2 and did not find any change in their levels after rotenone exposure, suggested that Miro1 and TNF-a/NF-kB/TNFaIP2 signaling pathways were not involved in our model. Furthermore, we also did not see any significant change in mitochondria uptake by neurons following the knocking down of Miro1 via Miro1 siRNA. Interestingly, photo-p38 protein levels were significantly elevated in the rotenone treated DA neurons, initially indicating the p38 MAPK signaling pathway may be involved in mitochondrial endocytosis in our case. In order to confirm this, we used a p38 inhibitor, SB203580, and observed that there was significantly less accumulation of astrocyte-derived mitochondria in the DA neurons. Furthermore, ACM from astrocytes did not rescue rotenone-damage DA neurons when the neurons were pre-exposed to SB203580. Those data demonstrated that the activated form of p38 involves in mitochondrial endocytosis, which contributed to the reconstruction of mitochondria function and the rescue of DA neurons.

## Conclusions

In summary, our studies showed that human iPSCs-derived astrocytes can act as mitochondrial donors supplying health mitochondria to rescue rotenone-induced DA neuron degeneration. In addition, the astrocytic mitochondria were internalized by the injured neurons via a phospho-p38 depended pathway. These findings provided a potential novel approach for PD treatment and a promising therapeutic target.

To this end, functional mitochondrial delivery by intracellular transfer presents a new paradigm of therapeutic intervention that benefits neuronal survival and regeneration for neurodegenerative diseases, stroke, and CNS injury.

## Supplementary information

**Additional file 1: Extended Fig. 1.** The NSCs were identification by immunocytochemistry (A) Representative images of SOX2 (red), and Nestin (green) positive (blue is DAPI) NSCs. Three independent experiments (*N* = 3). Scale bars represent 50 μm.

**Additional file 2: Extended Fig. 2.** Mitochondrial movement between astrocytes through TNTs like structure. (A) The representative immunofluorescence image of Blue-phalloidin-labeled F-actin astrocytes. The morphology of astrocytes and the TNTs like connections were outlined. (B) The Mito-Tracker Green labeled mitochondria exist among astrocytes and TNTs like structure. Three independent experiments (*N* = 3). Scale bars in all panels represent 20 μm.

**Additional file 3: Extended Fig. 3.** Activation of effective mitochondrial transfer following Rotenone exposure to DA neurons. (A) Shows the percentage of astrocytic mitochondria labeled with Mito-Tracker Green in ACM analyzed by flow cytometry. The assessment was performed under three different conditions: fresh ACM collected 24 h after complete media change (ACM), healthy neurons in ACM culture media with for 24 h (N + ACM), and rotenone treated neurons in ACM culture for 24 h (N + rot+ACM). In the fresh ACM, mitochondria account for 39.19% ± 0.98% of the total particles. In ACM that was in contact with healthy DA neurons, the mitochondrial decrease to 27.62% ± 1.30% of total particles, indicating that health DA neurons could intake mitochondria from media. However, in the ACM in contact with injured DA neurons (exposed to rotenone), the mitochondria reduced to 20.77% ± 2.09%, suggesting an increase in the uptake of mitochondrial from the extracellular medium. (B) Quantification of mitochondrial transfer ratio in the three conditions. Three independent experiments (*N* = 3).

**Additional file 4: Extended Fig. 4.** The knockdown of Miro 1 didn’t affect the mitochondrial transfer (A, B) Miro, I protein levels was significantly knocked down in DA neurons transfected with Miro 1 siRNA as shown by western blot of samples collected24h after transfection. (C) Flow cytometric analysis was used to determine the mitochondrial uptake efficiency. The Mito-Tracker Green positive neurons account for 19.36% of the total cells, similar to the percentage when Miro 1 was normal (see main Fig. 3b). Three independent experiments (*N* = 3). Results were presented as mean + SEM. * *P* < 0.05.

## Data Availability

All data generated or analyzed during this study are included in this published article.

## Abbreviations

PD: Parkinson’s disease
DA neurons: Dopaminergic neurons
ACM: Astrocytic conditioned media
dmACM: Mitochondria depleted ACM
LB: Lewy bodies
ATP: Adenosine triphosphate
OCR: Oxygen consumption rate
SN: Substantia nigra
ROS: Reactive oxygen species
OS: Oxidative stress
PMD: Peptide-mediated allogeneic mitochondrial delivery
MSCs: Mesenchymal stem cells
NSCs: Neural stem cells
TH: Tyrosine hydroxylase
Tuj1: Class III beta-tubulin
GFAP: Glial fibrillary acidic protein
TNTs: Tunneling nanotubes
MIRO1: Rho-GTPase 1 protein
