# Supplementary figures and images for: Human iPSCs derived astrocytes rescue rotenone-induced mitochondrial dysfunction and dopaminergic neurodegeneration in vitro by donating functional mitochondria
Source: Transl Neurodegener. 2020 Apr 24;9:13. doi: 10.1186/s40035-020-00190-6 (PMC7325238; doi:10.1186/s40035-020-00190-6)

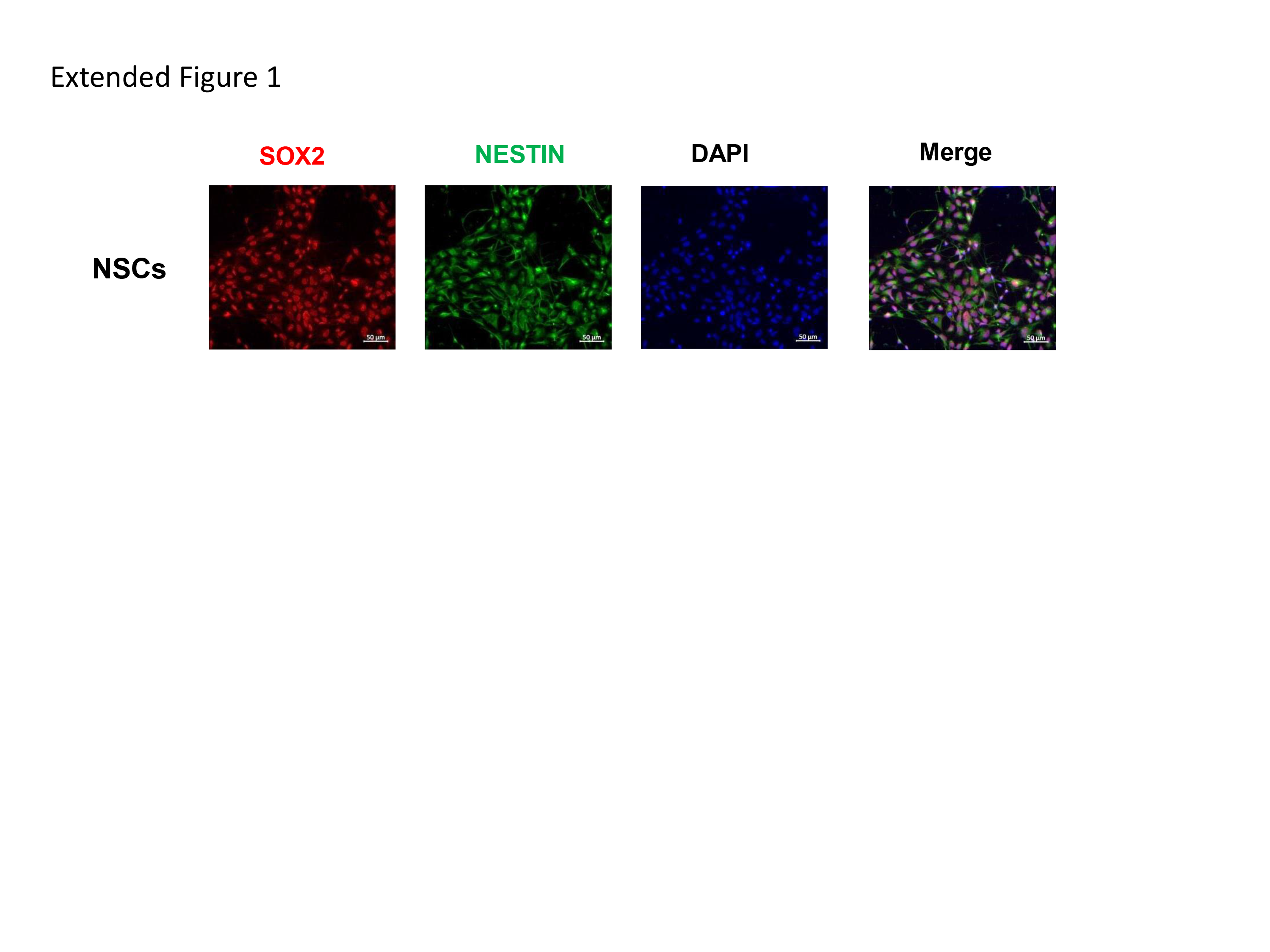

Supplement: Supplementary file 1 — Additional file 1: Extended Fig. 1. The NSCs were identification by immunocytochemistry (A) Representative images of SOX2 (red), and Nestin (green) positive (blue is DAPI) NSCs. Three independent experiments (N = 3). Scale bars represent 50 μm. [file 40035_2020_190_MOESM1_ESM.tif]

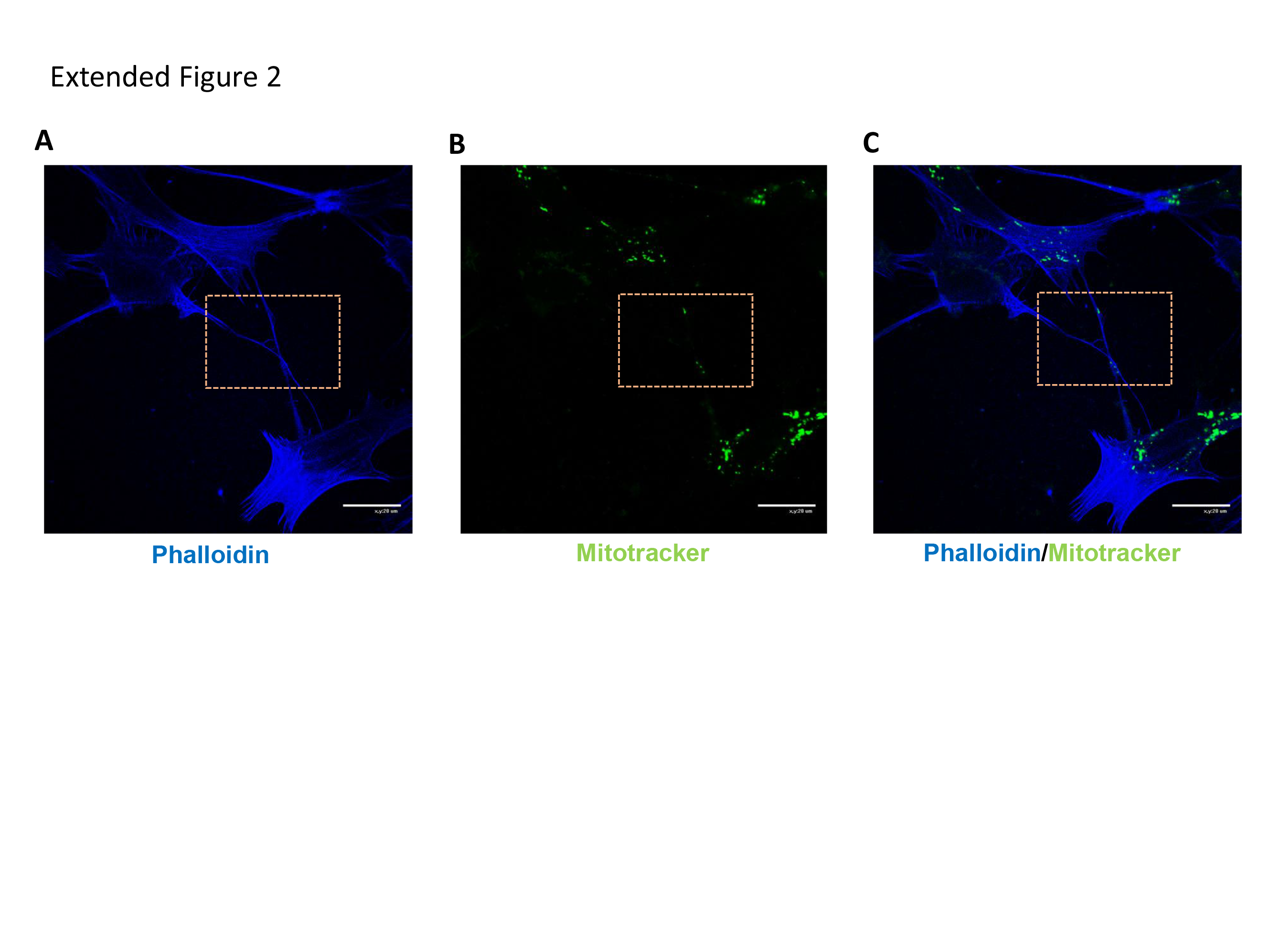

Supplement: Supplementary file 2 — Additional file 2: Extended Fig. 2. Mitochondrial movement between astrocytes through TNTs like structure. (A) The representative immunofluorescence image of Blue-phalloidin-labeled F-actin astrocytes. The morphology of astrocytes and the TNTs like connections were outlined. (B) The Mito-Tracker Green labeled mitochondria exist among astrocytes and TNTs like structure. Three independent experiments (N = 3). Scale bars in all panels represent 20 μm. [file 40035_2020_190_MOESM2_ESM.tif]

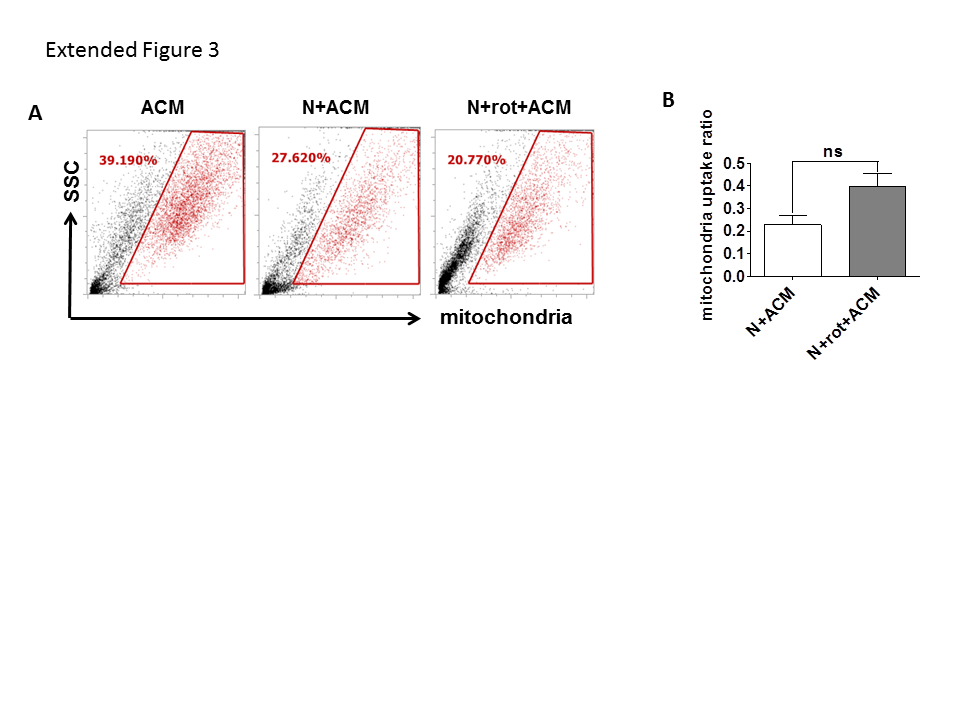

Supplement: Supplementary file 3 — Additional file 3: Extended Fig. 3. Activation of effective mitochondrial transfer following Rotenone exposure to DA neurons. (A) Shows the percentage of astrocytic mitochondria labeled with Mito-Tracker Green in ACM analyzed by flow cytometry. The assessment was performed under three different conditions: fresh ACM collected 24 h after complete media change (ACM), healthy neurons in ACM culture media with for 24 h (N + ACM), and rotenone treated neurons in ACM culture for 24 h (N + rot+ACM). In the fresh ACM, mitochondria account for 39.19% ± 0.98% of the total particles. In ACM that was in contact with healthy DA neurons, the mitochondrial decrease to 27.62% ± 1.30% of total particles, indicating that health DA neurons could intake mitochondria from media. However, in the ACM in contact with injured DA neurons (exposed to rotenone), the mitochondria reduced to 20.77% ± 2.09%, suggesting an increase in the uptake of mitochondrial from the extracellular medium. (B) Quantification of mitochondrial transfer ratio in the three conditions. Three independent experiments (N = 3). [file 40035_2020_190_MOESM3_ESM.tif]

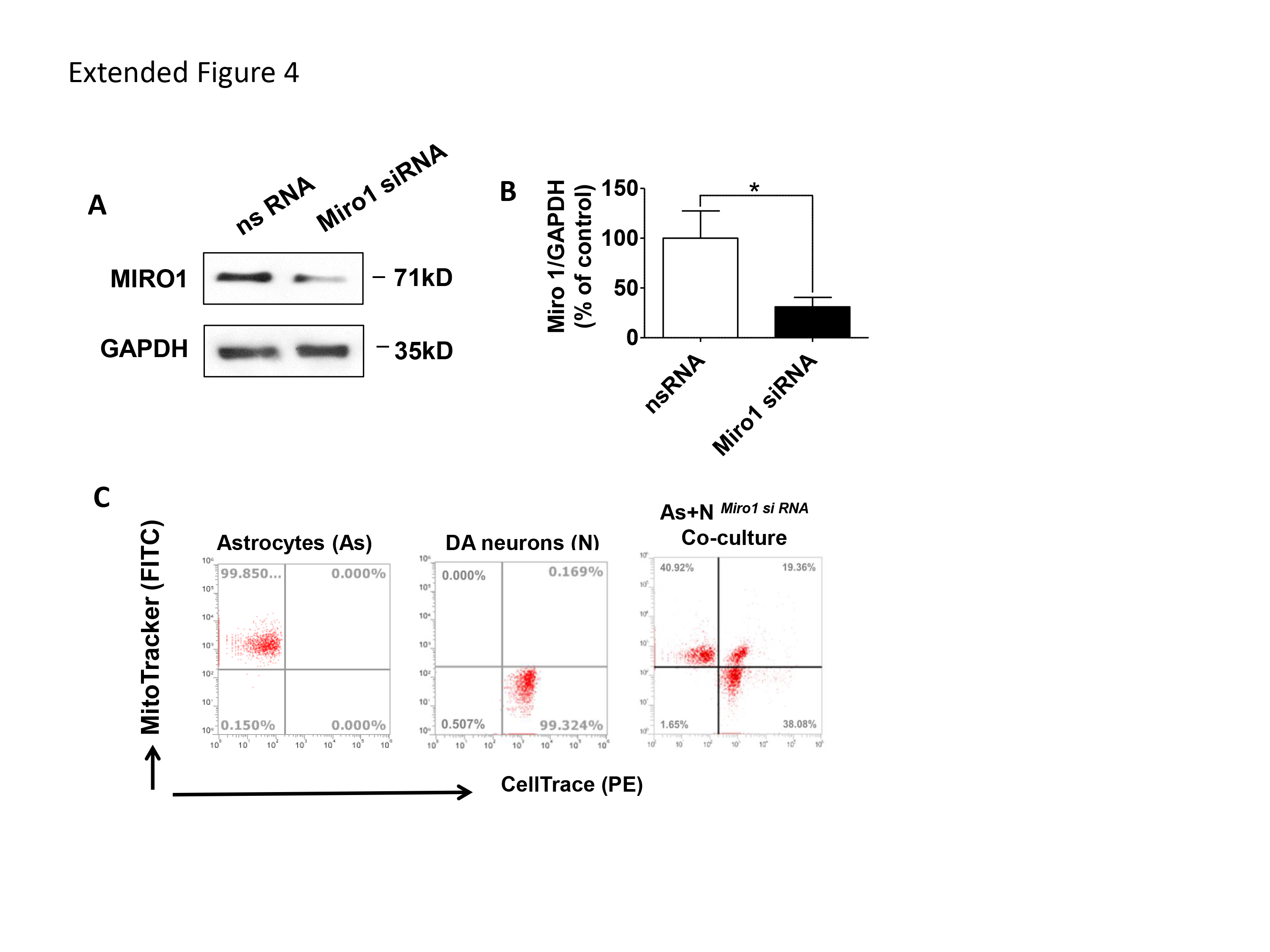

Supplement: Supplementary file 4 — Additional file 4: Extended Fig. 4. The knockdown of Miro 1 didn’t affect the mitochondrial transfer (A, B) Miro, I protein levels was significantly knocked down in DA neurons transfected with Miro 1 siRNA as shown by western blot of samples collected24h after transfection. (C) Flow cytometric analysis was used to determine the mitochondrial uptake efficiency. The Mito-Tracker Green positive neurons account for 19.36% of the total cells, similar to the percentage when Miro 1 was normal (see main Fig. 3b). Three independent experiments (N = 3). Results were presented as mean + SEM. * P < 0.05. [file 40035_2020_190_MOESM4_ESM.tif]
